# 3D morphometric analysis of fossil canid skulls contradicts the suggested domestication of dogs during the late Paleolithic

**DOI:** 10.1038/srep08299

**Published:** 2015-02-05

**Authors:** Abby Grace Drake, Michael Coquerelle, Guillaume Colombeau

**Affiliations:** 1 Department of Biology, Skidmore College, 815 North Broadway, Saratoga Springs, NY 12866; 2 Department of Paleobiology, Museo Nacional de Ciencias Naturales (MNCN-CSIC), C/Pinar 25, 28006 Madrid, Spain; 3 Department of Oral Surgery, University Rey Juan Carlos, Avda. de Atenas s/n, 28922-Alcorcón (Madrid), Spain; 4 UMR 5199 PACEA, Université de Bordeaux, Avenue des Facultés B18, F-33405 Talence CEDEX, France

## Abstract

Whether dogs were domesticated during the Pleistocene, when humans were hunter-gatherers, or during the Neolithic, when humans began to form permanent settlements and engage in agriculture, remains controversial. Recently discovered Paleolithic fossil skulls, Goyet dated 31,680 +/− 250 YBP and Eliseevichi MAE 447/5298 dated 13,905 +/− 55 YBP, were previously identified as dogs. However, new genetic studies contradict the identification of these specimens as dogs, questioning the validity of traditional measurements used to morphologically identify canid fossil skulls. We employ 3D geometric morphometric analyses to compare the cranial morphology of Goyet and Eliseevichi MAE to that of ancient and modern dogs and wolves. We demonstrate that these Paleolithic canids are definitively wolves and not dogs. Compared to mesaticephalic (wolf-like breeds) dog skulls, Goyet and Eliseevichi MAE, do not have cranial flexion and the dorsal surface of their muzzles has no concavity near the orbits. Morphologically, these early fossil canids resemble wolves, and thus no longer support the establishment of dog domestication in the Paleolithic.

The domestication of dogs is a significant event in the evolution of our species and the date and location of this event continue to be debated on both genetic and morphological fronts^1^,^2^,^3^,^4^,^5^,^6^,^7^,^8^,^9^,^10^,^11^,^12^,^13^,^14^. Germonpré *et al*.^10^ and Sablin and Khlopachev^11^ contend that the Goyet (31,680 +/− 250 YBP) and Eliseevichi MAE 447/5298 (13,905 +/− 55 YBP; Epigravettian) canid skulls are pre-Neolithic dogs, pushing the date of domestication back 15,000 years into the Paleolithic. Recently, Boudadi-Maligne and Escarguel^1^ found that Goyet did not fall within the range of size variability for dogs, however their sample composition was biased in that they only included small dogs for comparison and the Goyet specimen is large. Most importantly, all of these caliper-based morphometric analyses of fossil canids^1^,^3^,^6^,^8^,^10^,^11^,^12^,^14^ depend mainly on skull lengths and widths and many of these measurements overlap, which can lead to correlation amongst traits and contribute to conflicting results. Only Benecke^14^ included the third dimension of skull height, which varies substantially amongst domestic dogs^15^. Some researchers use ratios (e.g. palate length to total skull length) to control for size variation. However, as Wayne^16^ pointed out, nearly all canid skull length ratios are isometric and therefore do not provide shape information. Width to size ratios can discriminate some dogs from wolves, however these ratios are often combined with length ratios in multivariate analyses^6^,^10^, the results of which are then mostly comprised of size variation. Furthermore, it has been shown that using ratios in principal component analysis (PCA) is problematic because there are spurious correlations between ratios and their distribution is non-normal^17^. Therefore, previous statistical analyses that have attempted classification of fossil skulls based on these measurements^1^,^3^,^6^,^8^,^10^,^11^,^12^,^14^ should be reanalysed with more accurate methods.

Based on their canid mitochondrial genome study, Thalmann *et al*.^4^ conclude a European origin of the domestic dog dating as early as 18,800 to 32,100 years ago. Yet their molecular dating was calibrated with ancient canids, including Goyet and Eliseevichi MAE, whose identification was based on caliper measurements and ratios which have low resolution and do not distinguish dogs from wolves.

We employ 3D geometric morphometric methods which have been shown to provide powerful taxonomic assessment in other biological systems^18^,^19^,^20^ to reanalyse the skulls of Goyet and Eliseevichi MAE and compare them to skulls of ancient and modern dogs and wolves.

## Results

### Caliper Measurement Analysis

**Figure 1:**
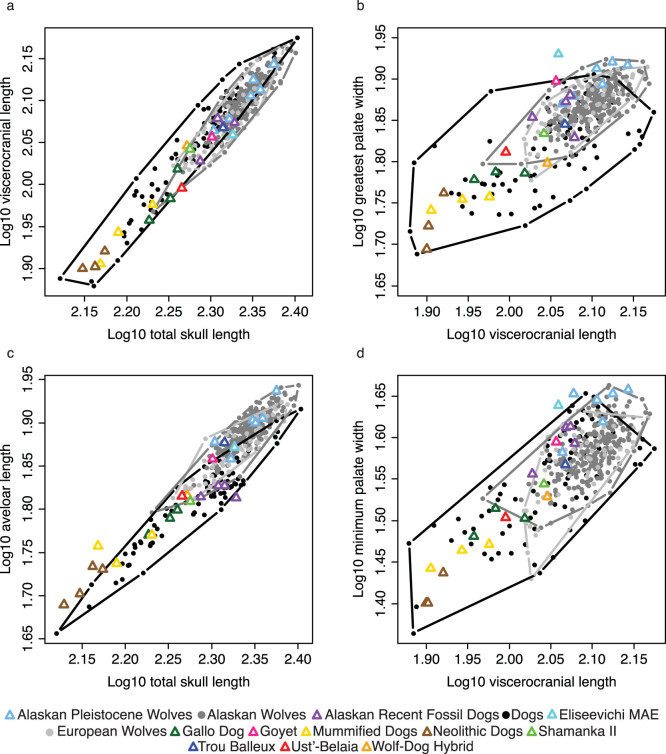
Bivariate plots of Euclidean distance based cranial indices. (a) Log_10_ total skull length versus Log_10_ viscerocranial length, (b) Log_10_ viscerocranial length versus Log_10_ greatest palate width (c) Log_10_ total skull length versus Log_10_ alveolar length, (d) Log_10_ viscerocranial length versus Log_10_ minimum palate width. Convex hulls of dogs, Alaskan wolves, and European wolves are outlined.

**Figure 2:**
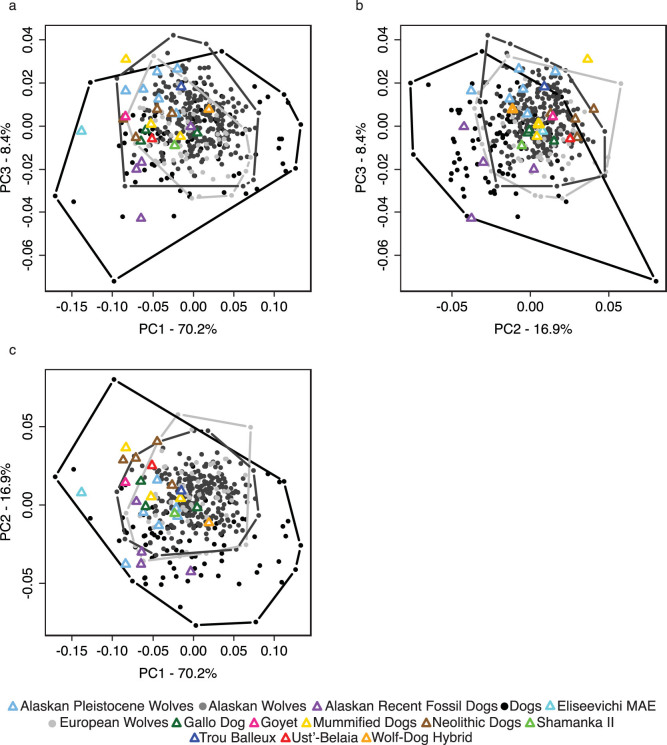
PCA of Euclidean distance based cranial indices. (a) PC1 versus PC3, (b) PC2 versus PC3, (c) PC1 versus PC2. Convex hulls of dogs, Alaskan wolves, and European wolves are outlined.

**Figure 3:**
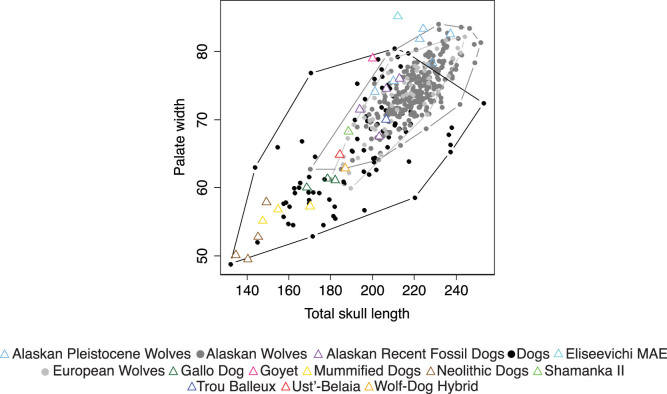
Bivariate plot of palate width versus total skull length. Convex hulls of dogs, Alaskan wolves, and European wolves are outlined.

Bivariate plots of linear distances (Fig. 1) and PCA of cranial ratios (Fig. 2) demonstrate that there is nearly complete overlap of dogs and wolves making any diagnostic criterion of phenotypic differences impossible. Because these measurements and analyses are insufficient for detecting morphological differences between modern dogs and wolves they should not be used when classifying fossil specimens. Boudadi-Maligne and Escarguel's^1^ analysis included only very small archaeological dogs such that the large Goyet skull was not compared to dogs of a similar size. Figure 3 clearly shows that there is no separation of dogs and wolves in a comparison of palate width to total skull length unless only very small dogs are included.

### Geometric Morphometric Analysis

**Figure 4:**
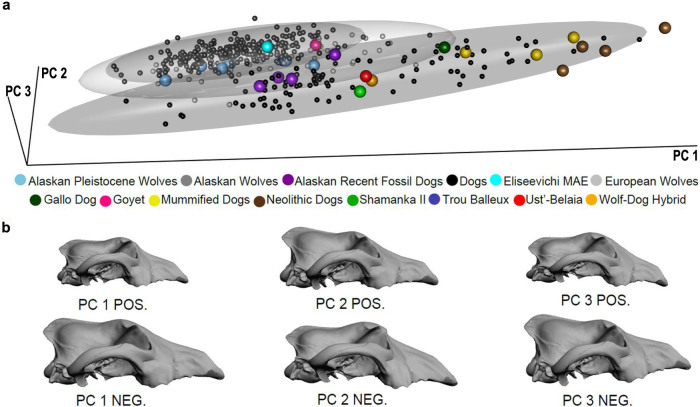
PCA plot of 36 form space coordinates. (a) 3D plot of PC1, PC2 and PC3, (b) 3D morphs of extremes along PC 1, PC 2 and PC 3. 95% Confidence interval ellipsoids of modern dogs, Alaskan wolves, and European wolves are outlined. Unclassified specimens are labelled separately in this and other figures. A 3D version of this figure is available as Supplementary Figure S1.

The first three principal components (PCs) of the Procrustes form space PCA using only modern dogs and wolves account for 88% of the total form variance (Fig. 4, a). Although wolves have, on average, larger skulls than dogs (P < 0.001; permutation test, n = 1,000), they overlap along PC1, which characterizes overall size variation as well as static allometry (r = 0.99). Eurasian wolves display greater variation along PC1 than Arctic wolves due to the greater amount of size variation in this subspecies (P < 0.0001; permutation test, n = 1,000). Dogs and wolves separate better in the subspace formed by PC2 and PC3, which accounts for size-independent shape variation (r_PC2_ = −0.01, r_PC3_ = 0.002). The fossils Goyet, Eliseevichi MAE, Trou Balleux, and five of the Pleistocene Arctic wolves lie exclusively within the wolf shape variation, whereas one Pleistocene Arctic wolf and one of the recent fossil Alaskan dogs, are positioned in the morphospace shared by dogs and wolves. The wolf-dog hybrid, all of the Neolithic dogs including the Ust'-Belaia and Shamanka II^21^, and the mummified dogs fall entirely within the dog shape variation.

**Figure 5:**
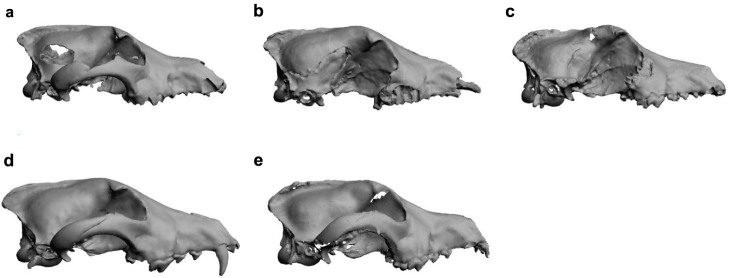
3D surface models of fossil specimens used in this analysis. (a) Eliseevichi MAE 447/5298, (b) Goyet, (c) Shamanka II, (d) Trou Balleux, (e) Ust'-Belaia. A 3D version of this figure is available as Supplementary Figure S2.

The shape variation at the negative end of PC2, where the dogs are located, shows that the nasal bones are shifted relatively posteriorly while the frontal bones are shifted relatively anteriorly, creating the angle between the forehead and the muzzle known as the “stop” (Fig. 4, b, PC2 NEG). This is in contrast to the flatter shape of the wolf skull where the top of the muzzle shows no concavity (Fig. 4, b, PC2 POS). The Eliseevichi MAE, Goyet, and Trou Balleux canid skulls share with wolves a lack of a pronounced stop (Fig. 5, a, b, d). On the other hand, these dog-like characteristics are clearly seen in the Neolithic specimens, Shamanka II and Ust'-Belaia^21^ (Fig. 5, c, e).

The resampling procedure involving one-thousand iterations of a cross-validation Quadratic Discriminant Analysis (QDA) to ensure equal wolf and dog sample sizes, correctly classifies 96% of the modern skulls with a posterior probability *P_post_* > 0.90 (Tau = 0.916, Wilks' lambda = 0.150). This result allows us to distinguish between dogs and wolves with considerable certainty, and therefore to test the categorization of the fossil skulls to either group. This analysis confirms that the fossils Eliseevichi MAE, Goyet, and Trou Balleux are classified as wolves as were four of the six Pleistocene Arctic wolf fossils with a *P_post_* > 0.90 (Table 1, a). Moreover, the typical probability of each of these fossils as belonging to the wolf group supports the QDA classification (Table 1, a). Given that 63 out of the 1000 iterations (6.3%) of the typical probabilities contradict the QDA classification of Shamanka II as either a wolf or dog indicates this specimen may be a hybrid and warrants further investigation. Ust'-Belaia, the Gallo-Roman canid, the wolf-dog hybrid, and all of the other Neolithic and mummified dogs are classified as dogs with a *P_post_* > 0.90 and this classification is further supported by the typical probabilities (Table 1, a).

**Figure 6:**
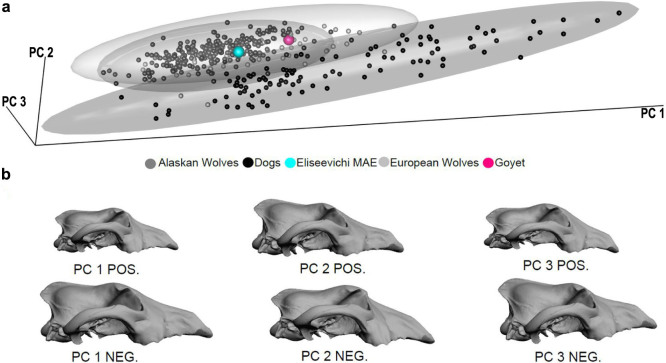
PCA plot of 36 form space coordinates including classified fossil specimens. (a) 3D plot of PC1, PC2 and PC3, (b) 3D morphs of extremes along PC 1, PC 2 and PC 3. 95% Confidence interval ellipsoids of modern dogs, Alaskan wolves and European wolves are outlined. Unclassified specimens are labelled separately in this and other figures. A 3D version of this figure is available as Supplementary Figure S3.

We repeated our entire analysis with all fossils in their respective groups except for Goyet and Eliseevichi MAE. The two Pleistocene Arctic wolves that were undetermined were not included. The first three PCs capture 90% of the total form variance and display the same set of shape variations as the previous PCA with only modern dogs and wolves (Fig. 6). The fossils Goyet and Eliseevichi MAE are still positioned within the wolf shape variation indicating that the addition of the fossil wolves and dogs to the analysis does not change the classification of these Paleolithic canids as wolves.

The performance of the cross-validation QDA of the first six PCs, accounting for 93% of the total form variance, correctly classify 96% of wolf and dog skulls *P_post_* > 0.90 (Tau = 0.919, Wilks' lambda = 0.196). The Goyet and Eliseevichi MAE skulls were classified as wolves in 100% of the 1,000 iterations of the resampling procedure and their typicality probabilities support this classification (Table 1, b).

## Discussion

Based on caliper measurements and distance ratios, the fossil skulls Goyet (31,680 +/− 250 YBP) and Eliseevichi MAE (13,905 +/− 55 YBP) were previously identified as dogs, establishing the date of dog domestication in the Paleolithic contemporaneous with human hunter-gatherers^6^,^10^,^11^. Our analysis shows that these measurements do not provide adequate resolution for distinguishing dogs from wolves in comparison to 3D landmark-based geometric morphometric methods. Geometric morphometric methods preserve size and shape information and allow the inclusion of shape variation that cannot be gathered via calipers measurements. Our geometric morphometric study demonstrates that the fossil canids Goyet and Eliseevichi MAE are wolves and hence contradicts the establishment of dog domestication in the Paleolithic based on these two specimens.

Dogs differ from wolves in various ways. All breeds display some degree of cranial flexion, most breeds have a dorsally angled muzzle and shortening of the nasal bones while a few breeds have a ventrally angled muzzle^15^,^22^. Those breeds with a muzzle that is flexed dorsally often have a marked stop where the muzzle meets the braincase. In breeds where the stop is not pronounced, there is still a forward projection of the frontals which angles the orbits vertically on the skull in addition to an elevated muzzle and shortened nasals. Previous studies have demonstrated the relative modularity of the face and neurocranium in carnivores, wolves and dogs^15^,^22^. This modularity has a phylogenetic history and a developmental basis which allowed for the cranial flexion that distinguishes dogs from wolves^15^,^22^.

Recent analysis of complete mitochondrial genomes revealed that Goyet, and other Paleolithic wolves, belong to a sister clade to all ancient and modern dogs^4^. In addition, Eliseevichi MAE, which was found in Russia, is not found in a clade with modern dogs but is instead genetically affiliated with modern wolves from Finland and Russia^4^. Our Procrustes form analysis is in accordance with this genetic evidence. Goyet and Eliseevichi MAE lie within the wolf morphospace, together with the Paleolithic Alaskan wolves and Trou Balleux from Belgium. The form of these specimens is definitively similar to neither modern nor to ancient dogs. Therefore, a reassessment of the classification of the other fossil canids such as the Altai specimen using 3D landmark-based geometric morphometric methods combined with genetic data, is needed to address the origin of domestication.

Alone, our new classification of Goyet and Eliseevichi MAE as wolves, supports a reestablishment of the timing of dog domestication in the Neolithic. Coppinger and Coppinger^23^ hypothesized that dog domestication occurred during the Neolithic when wolves began to scavenge near human settlements. Their assumption was that human settlements provided a new niche because of the permanent supply of waste food. Belyaev's^24^,^25^ experiment with silver foxes (*Vulpes vulpes*) clearly shows how domestication could take place quickly once a food source, that would increase fitness for wolves that could access it, was readily available. Belyaev selected wild silver foxes (which are typically anxious and aggressive) for tameness. Within only a few generations the offspring of the selected foxes showed no fear of humans and would even engage in care-soliciting behaviour. Remarkably, by the twentieth generation the foxes also had many other traits that are associated with domestication such as floppy ears and pie-bald coats^24^,^25^. The establishment of permanent settlements in the Neolithic would have created an environment where sustained selection for tameness could exist for many generations thus setting the stage for dog domestication.

## Methods

### Comparative sample of adult modern dogs and wolves

We carefully chose only dogs (N = 91) whose breeds closely resemble wolves in skull shape (for a complete list of breeds used see Supplementary Note S1). The wolf sample is composed of Arctic wolves (*Canis lupus arctos*) (N = 258) from Alaska and European wolves (*Canis lupus lupus*) (N = 57). For modern dog and wolf specimen locations see Supplementary Note S1.

### Sample of ancient canids

In addition to Eliseevichi MAE 447/5298 (13,905 +/− 55 YBP; Epigravettian)^11^ and Goyet (31,680 +/− 250 YBP)^10^ we include in our fossil sample of ancient canids Shamanka II (7,372 +/− 47 YBP) and Ust'-Belaia (6,817 +/− 63 YBP)^21^, which were found in the Lake Baikal region of Eastern Siberia and identified as early Neolithic dogs, Trou Balleux which was previously identified as a late Paleolithic wolf (10,110 +/− 120 YBP)^26^ from Belgium, six Alaskan wolf skulls from the late Pleistocene and beginning of Holocene, and four ancient Alaskan dog skulls dated to near 1600 CE, deposited before the first arrival of European explorers^27^, three Egyptian mummified dogs from the Saite–Ptolemaic period^28^, four Neolithic and one Gallo-Roman dog from France^29^, and a modern wolf-dog hybrid (Supplementary Table S1 and Supplementary Note S1).

### Digitization of the 3D anatomical landmarks

We captured the 3D coordinates from 36 osteological landmarks (descriptions given in Supplementary Table S2) on the dorsal and ventral surfaces of the skulls. The dorsal and ventral coordinate configurations were combined into one set of coordinates using a least-squares fit (rotation and translation only) of four matching landmarks^15^,^22^. Each skull was digitized twice in order to quantify measurement error.

### Cranial Index Analysis

Using the coordinate data we calculated Log_10_ total skull length, Log_10_ viscerocranial length, Log_10_ alveolar (P4-M1) tooth row length, Log_10_ greatest palate width and Log_10_ minimum palate width for all specimens. These measurements were used to calculate the following cranial indices: Log_10_ viscerocranial length/Log_10_ total skull length, Log_10_ alveolar (P4-M1) tooth row length/Log_10_ total skull length, Log_10_ greatest palate width/Log_10_ viscerocranial length, and Log_10_ minimum palate width/Log_10_ viscerocranial length following Germonpre *et al*^10^. We conducted a principal components analysis (PCA) of the wolf and dog cranial indices. Eliseevichi MAE, Goyet, Shamanka II, Trou Balleux, Ust'-Belaia and the other fossil wolf and dog specimens (see Supplementary Table S1) were then projected into the wolf-dog cranial index PCA. We also constructed bivariate plots of these indices to illustrate plainly the overlap of dogs and wolves. In addition, we recreated Boudadi-Maligne and Escarguel's^1^ plot of palatal width versus total skull length again to show how, when large dogs are included, there is overlap of dogs and wolves in this morphospace (Note: Boudadi-Maligne and Escarguel's^1^ use condyobasal length (Prosthion to the Occipital Condyles) – because we lack the point on the condyles we used total skull length (Prosthion to Basion) as a very close approximation).

### Procrustes superimposition

Geometric morphometric analysis of three-dimensional landmark-based coordinates is an effective diagnostic tool for investigating biological shape that allows for the direct visualization of shape variation^15^,^22^,^30^,^31^,^32^,^33^,^34^. The raw coordinates of the landmark-based configurations of the canid skulls were converted to shape coordinates by generalized least-squares Procrustes superimposition using a procedure that takes into account the object symmetry of the specimens^32^,^35^. This involves rescaling the landmark coordinates so that each configuration has a unit Centroid Size (CS: square root of the summed squared Euclidean distances from all landmarks to their centroid). Then all configurations were translated and rotated to minimize the overall sum of the squared distances between corresponding landmarks. The amount of measurement error was calculated using a Procrustes ANOVA and was found to be insignificant^35^; we therefore averaged all replicates into a single configuration for each specimen.

### Procrustes form space principal component analysis

A significant reduction of the overall size of the skulls is thought to characterize Paleolithic dog compared to Pleistocene wolf skulls^1^,^3^,^6^,^8^,^10^,^11^,^12^. Therefore, in addition to shape variables, a measure of overall size such as Centroid Size, for which the shape variables are independent, may help to determine whether fossil canids are either wolves or dogs. Centroid Size has been shown to be approximately uncorrelated with the shape variables for landmarks with small amounts of isotropic variation. After performing the Procrustes superimposition, size and shape variation were first explored with a PCA based on the covariance matrix of the dog and wolf Procrustes shape coordinates augmented by a column of the natural logarithm of Centroid Size (LnCS) – called Procrustes form space PCA^33^. The fossil specimens Eliseevichi MAE, Goyet, Shamanka II, Trou Balleux, Ust'-Belaia, and the fossils cited above (Supplementary Table S1) were projected into the wolf-dog Procrustes form space PCA. The original covariance matrix used for the PCA excludes the fossil data because we wanted to identify where the fossil canid skulls would plot in an ordination of known wolf and dog skulls and to show the range of variation in Procrustes form space spanned between extant and extinct canids. In Procrustes form space PCA, PC1 usually captures overall size variation as well as size-related shape variation (allometry), whereas the other PCs contain residual, non-allometric, shape variation and are weakly correlated with size.

A 3D digital scan of a wolf skull was warped towards the Procrustes mean form using a thin plate spline (TPS) interpolation function using IDAV Landmark software^34^. Thereafter, the surface of the Procrustes mean configuration (consensus) was used to visualize size and shape variation along the PCs. The shape deformation represented by the eigenvectors of a particular PC was visualized as a TPS deformation from the consensus *plus* or *minus* the eigenvectors (right and left side of the PC, respectively). Once the eigenvectors (those related to the shape variables) are added or subtracted from the consensus, all variables are also multiplied by the exponent of the eigenvector for LnCS.

### Quadratic discriminant analysis and typical probability

QDA with cross validation was used for the classification of the unknown specimens Eliseevichi MAE, Goyet, Shamanka II, Trou Balleux, and Ust'-Belaia as well as the fossil specimens cited above (Supplementary Table S1). The use of QDA is justified because the Box's M test indicates that the dog and wolf covariance matrices are significantly different (Mbox = 253.94, df = 28, p-value <0.001).

Because we limited the types of breeds included in this analysis the wolf sample size is more than three times larger than that of the dogs. In order to balance the wolf and dog sample sizes, we randomly resampled the wolf specimens to create a dataset of 91 specimens. We then carried out a Procrustes form space PCA of the two groups (without the fossil skulls). This was done 1,000 times. Using a statistical test borrowed from Anderson^36^,^37^,^38^, for each iteration, we found that the eigenvalues from PC8 onwards were nearly equal and hence their ordination is more likely than not to be expressing only noise. Therefore, for each iteration we built a QDA model based on the first seven PCs of the Procrustes form space PCA accounting for 93% of the total form variance (as much as in the original Procrustes form space PCA with the full wolf and dog sample). The computation of the posterior probabilities (*P_post_*) was made with an equal prior probability (*P_prior_* = 0.5) for the dogs and wolves. We assigned specimens to either the dog or wolf group only if the *P_post_* was greater than 0.90. The posterior probabilities are the probability of membership for each specimen in each group based on the relative distances to each group, and they sum to 1. Therefore, the unknown specimens are forced to belong to one of the reference groups. Because of this, we defined a threshold of correct classification, giving the unknown specimen the opportunity to belong to neither of the reference groups, *i.e.* to be classified as an undetermined specimen (*P_post_* < 0.90). Each unknown specimen's cranium was tested through all 1,000 iterations of the cross-validation QDAs. The accuracy of the classification was computed as the percentage of iterations for which the unknown specimen was classified with a *P_post_* > 0.90 either as dog, wolf or unknown.

We also computed each specimen's typical probability (Typ.P) in order to evaluate the fit of a specimen to a group^39^. This probability represents how likely an unknown skull belongs to a particular group based on the variance-covariance matrix of the wolf and dog data pooled together. This probability is analogous to the probability of the null hypothesis that the specimen comes from a particular group. If above 0.05 the typical probability can be ignored, because there is no statistical ground to reject the null hypothesis. However if Typ.P ≤ 0.05, then the specimen's posterior probability should be ignored because the specimen does not belong to either the wolf or dog group.

We wanted to know whether including fossil specimens in the dog and wolf groups would change the classification of Eliseevichi MAE and Goyet. Therefore, after classifying the fossil specimens as either dogs or wolves based on the above analysis, we repeated the entire analysis with only Eliseevichi MAE and Goyet as unknowns. In this analysis we found that the eigenvalues from PC7 onwards were nearly equal. We therefore built the QDA model based on the first six PCs of the Procrustes form space PCA accounting for 93% of the total form variance for each iteration. All data were analyzed via software routines written in the R programming language.

## Supplementary Material

Supplementary Information Supplementary Information

Supplementary Information Figure S1

Supplementary Information Figure S2

Supplementary Information Figure S3

## Figures and Tables

**Table 1:** Results of the resampling procedure for the QDA

a. PCs 1–7; 36 landmarks								
	Average Ppost		Percentage of iterations for which specimen's Ppost ≥0.90			QDA Result	Typical Probability	
Specimen	Dog	Wolf	Dog	Wolf	Ind	Group	%Typ.P ≤ 0.05	Group
Eliseevichi MAE 447/5298	0.01	0.99	0.1	98.6	1.4	Wolf	0.0	Wolf
Goyet	0.00	1.00	0.0	100.0	0.0	Wolf	0.0	Wolf
Shamanka II	1.00	0.00	100.0	0.0	0.0	Dog	6.3	Dog
Trou Balleux	0.00	1.00	0.0	100.0	0.0	Wolf	0.0	Wolf
Ust'-Belaia	1.00	0.00	100.0	0.0	0.0	Dog	0.0	Dog
NHML 51.000.078	1.00	0.00	100.0	0.0	0.0	Dog	0.0	Dog
INRAP FO3257	1.00	0.00	100.0	0.0	0.0	Dog	0.0	Dog
NHML 51.000.020	1.00	0.00	100.0	0.0	0.0	Dog	0.0	Dog
NHML 51.000.022	1.00	0.00	100.0	0.0	0.0	Dog	0.0	Dog
NHML 51.000.023	1.00	0.00	100.0	0.0	0.0	Dog	3.9	Dog
MLS 1040	1.00	0.00	100.0	0.0	0.0	Dog	0.9	Dog
MLS 726	1.00	0.00	100.0	0.0	0.0	Dog	0.0	Dog
MLS 718	1.00	0.00	100.0	0.0	0.0	Dog	0.0	Dog
MLS 621	1.00	0.00	100.0	0.0	0.0	Dog	0.0	Dog
AMNH 30435	1.00	0.00	100.0	0.0	0.0	Dog	0.0	Dog
AMNH 30436	0.99	0.01	99.0	0.0	0.1	Dog	0.0	Dog
AMNH 67155a	1.00	0.00	100.0	0.0	0.0	Dog	0.0	Dog
AMNH 70932	0.99	0.01	99.5	0.0	0.5	Dog	0.0	Dog
AMNH 30450	0.00	1.00	0.0	100.0	0.0	Wolf	0.0	Wolf
AMNH 30433	0.10	0.90	0.6	78.4	21.0	Ind	0.0	Wolf
AMNH 67157	0.32	0.68	4.2	23.3	72.5	Ind	0.0	Wolf
AMNH 97079	0.01	0.99	0.1	98.8	1.1	Wolf	0.0	Wolf
AMNH 30431	0.00	1.00	0.0	100.0	0.0	Wolf	0.0	Wolf
AMNH 67163	0.00	1.00	0.0	100.0	0.0	Wolf	0.0	Wolf

b. PCs 1–6; 36 landmarks, with fossils included in the wolf and dog samples								
	Average Ppost		Percentage of iterations for which specimen's Ppost ≥0.90			QDA Result	Typical Probability	
Specimen	Dog	Wolf	Dog	Wolf	Ind	Group	%Typ.P ≤ 0.05	Group
Eliseevichi MAE 447/5298	0.01	0.99	0.1	98.4	1.5	Wolf	0.0	Wolf
Goyet	0.00	1.00	0.0	100.0	0.0	Wolf	0.0	Wolf
