# Supplementary material for: 3D morphometric analysis of fossil canid skulls contradicts the suggested domestication of dogs during the late Paleolithic
Source: Sci Rep. 2015 Feb 5;5:8299. doi: 10.1038/srep08299 (PMC5389137; doi:10.1038/srep08299)
Supplement: Supplementary Information [file srep08299-s1.pdf]

Supplementary Information for: **“**3D morphometric analysis of fossil canid skulls contradicts the suggested domestication of dogs during the late Paleolithic”

Abby Grace Drake[1,]* Michael Coquerelle[2,3] Guillaume Colombeau[4]

Supplementary Figure S1. **PCA plot of 36 form space coordinates.** (a) 3D plot of PC1, PC2 and PC3, (b) 3D morphs of extremes along PC 1, PC 2 and PC 3. 95% Confidence interval ellipsoids of modern dogs, Alaskan wolves, and European wolves are outlined. Unclassified specimens are labelled separately in this and other figures.To view and interact with this 3D figure download the pdf and open it in Adobe Acrobat.

Supplementary Figure S2. **3D surface models of fossil specimens used in this analysis.** (a) Eliseevichi MAE 447/5298, (b) Goyet, (c) Shamanka II, (d) Trou Balleux, (e) Ust’-Belaia**.** To view and interact with this 3D figure download the pdf and open it in Adobe Acrobat.

Supplementary Figure S3. **PCA plot of 36 form space coordinates including classified fossil specimens.** (a) 3D plot of PC1, PC2 and PC3, (b) 3D morphs of extremes along PC 1, PC 2 and PC 3. 95% Confidence interval ellipsoids of modern dogs, Alaskan wolves and European wolves are outlined. Unclassified specimens are labelled separately in this and other figures. To view and interact with this 3D figure download the pdf and open it in Adobe Acrobat.

Supplementary Note S1:

Breeds in this analysis include: Bernese Hound Dog, Bloodhound, Chesapeake Bay Retriever, Dalmatian, Dingo, German Shepherd, Greater Swiss Mountain Dog, Greenland Dog, Irish Wolfhound, Jura Laufhund, Jura Laufhund St. Hubert, New Guinea Singing Dog, Norwegian Puffin Dog, Pharoh Hound, Samojede, Shar Pei, Siberian Husky, Swedish Lapphund, Tervueren, and Wolfspitz.

Eliseevichi MAE 447/5298 (13,905 +/- 55 YBP; Epigravettian)[11], was found in the Bryansk Region of Russia and was originally identified as a dog based on caliper measurements because it has a very wide skull. The Goyet skull (31,680 +/- 250 YBP)[10] is from Goyet cave in Belgium and was identified as a Paleolithic dog by a multivariate analysis of width and length ratios of the skull. Our comparative sample of ancient canids include Shamanka II (7,372+/- 47 YBP) and Ust'-Belaia (6,817 +/- 63 YBP)[21], which were found in the Lake Baikal region of Eastern Siberia. These two specimens, were identified as early Neolithic dogs not only by their morphology but also by cultural signals. Both were found in graves and Ust'-Belaia "was interred wearing a necklace of eight red deer canine tooth pendants"[21]. We also incorporate the specimen Trou Balleux that was previously identified as a late Paleolithic wolf (10,110 +/- 120 YBP)[26] from Belgium via a multivariate analysis of width and length ratios of the skull[10]. We also included six Alaskan wolf skulls from the late Pleistocene and beginning of Holocene, which are concurrent with Goyet and Eliseevichi MAE. Finally, we included four ancient Alaskan dog skulls dated to near 1600 CE, deposited before the first arrival of European explorers[27], three Egyptian mummified dogs from the Saite–Ptolemaic period[28], four Neolithic and one Gallo-Roman dog skulls from France[29], and a modern wolf-dog hybrid (Supplementary Table S1).

The majority of dog specimens are housed in the Albert Heim Collection at the Natural History Museum in Berne, Switzerland. The majority of wolf specimens are from the University of Alaska Museum in Fairbanks, Alaska. Other specimens are from the Smithsonian Institution's National Museum of Natural History in Washington, DC, the Museum of Vertebrate Zoology at the University of California in Berkeley, California, and the Zoology Department at the Natural History Museum, London.

The CT-scans of Goyet, Eliseevichi MAE, Trou Balleux, Shamanka II and Ust'-Belaia were kindly provided by Mietje Germonpré, Royal Belgian Institute of Natural Sciences, Robert Losey, University of Alberta, and Mikhail Sablin, Zoological Institute of the Russian Academy of Sciences. The wolf-dog hybrid as well as the Egyptian dog mummies are housed in the Natural History Museum of Lyon, France. The French Neolithic dogs were provided by the Museum of Lons le Saunier and the Gallo-Roman dog was provided by the Institut National de la Recherche Archéologique Préventive of Aquitaine, France.

Supplementary Table S1:

**Specimens**	**Age**	**Culture**	**Origin**	**Previous Classification**
Eliseevichi MAE 447/5298	13,905 +/- 55 YBP[11]	Epigravettian	Russia	Dog
Goyet	31,680 +/- 250 YBP[10]	Late Paleolithic	Belgium	Dog

Shamanka II	7,372 +/- 47 YBP[21]	Neolithic	Siberia	Dog
Trou Balleux	10,110 +/- 120 YBP[10]	Final Paleolithic	Belgium	Wolf
Ust'-Belaia	6,817 +/- 63 YBP[21]	Neolithic	Siberia	Dog
NHML 51.000.078	-	Contemporaneous	France	Wolf-Dog Hybrid
INRAP FO3257	-	Gallo-Roman	France	Dog
NHML 51.000.020	800 - 100 YBP[28]	Saite - Ptolemaic	Egypte	Dog
NHML 51.000.022	800 - 100 YBP[28]	Saite - Ptolemaic	Egypte	Dog
NHML 51.000.023	800 - 100 YBP[28]	Saite - Ptolemaic	Egypte	Dog
MLS 1040	3,200-2,900 YBP[29]	Neolithic	France	Dog
MLS 726	3,200-2,900 YBP[29]	Neolithic	France	Dog
MLS 718	3,200-2,900 YBP[29]	Neolithic	France	Dog
MLS 621	3,200-2,900 YBP[29]	Neolithic	France	Dog
AMNH 30435	1665 CE[27]	Post-Classic	Alaska	Dog
AMNH 30436	1520, 1600, 1630 CE[27]	Post-Classic	Alaska	Dog
AMNH 67155a	1655 CE[27]	Post-Classic	Alaska	Dog
AMNH 70932	1640 CE[27]	Post-Classic	Alaska	Dog
AMNH 30450	8,455 +/- 59 YBP[27]	Archaic Stage	Alaska	Wolf
AMNH 30433	14,771 +/- 195 YBP[27]	Late Paleolithic	Alaska	Wolf
AMNH 67157	16,377 +/- 131 YBP[27]	Late Paleolithic	Alaska	Wolf
AMNH 97079	28,353 +/- 573 YBP[27]	Late Paleolithic	Alaska	Wolf
AMNH 30431	33,888 +/- 347 YBP[27]	Late Paleolithic	Alaska	Wolf
AMNH 67163	>37,183 YBP[27]	Late Paleolithic	Alaska	Wolf

Supplementary Table S2:

Landmark Number	Osteological Description
1	Midline point on the premaxilla at the inferior tip of the bony septum between upper central incisors.
2	Frontal-maxillary-nasal suture, left side.
3	Frontal-maxillary-nasal suture, right side.
4	Nasion, nasal-frontal suture, midline.
5	Frontal-maxillary suture, posterior, left side.
6	Frontal-maxillary suture, posterior, right side.
7	Frontal-parietal-sphenoid suture, left side.
8	Bregma, frontal-parietal suture, midline.
9	Frontal-parietal-sphenoid suture, right side.
10	Lambda, parietal-occipital suture, midline.
11	Asterion, posterior at occipital-parietal-temporal suture, left side.
12	Asterion, posterior at occipital-parietal-temporal suture, right side.
13	Opsithion, dorsal lip of foramen magnum, midline.
14	Occipital Condyle – widest point of foramen magnum, left side.
15	Occipital Condyle – widest point of foramen magnum, right side.
16	Basion, ventral lip of foramen magnum, midline.
17	External auditory meatus, posterior, left side.
18	External auditory meatus, posterior, right side.
19	Premaxillary-maxillary suture, posterior at midline.
20	Maxillary-palatine suture, anterior at midline.
21	Tympano-occipital fissure, anterior lip, right side.
22	Tympano-occipital fissure, anterior lip, left side.
23	Canine – posterior buccal corner, right side.
24	Premolar 1 – posterior buccal corner, right side.
25	Premolar 2 – posterior buccal corner, right side.
26	Premolar 3 – posterior buccal corner, right side.

27 Premolar 4 – posterior buccal corner, right side.

28 Molar 1 – posterior buccal corner, right side.

29 Molar 2 – posterior buccal corner, right side.

30 Canine – posterior buccal corner, left side.

31 Premolar 1 – posterior buccal corner, left side.

32 Premolar 2 – posterior buccal corner, left side.

33 Premolar 3 – posterior buccal corner, left side.

34 Premolar 4 – posterior buccal corner, left side.

35 Molar 1 – posterior buccal corner, left side.

36 Molar 2 – posterior buccal corner, left side.
