# Supplementary material for: 3D morphometric analysis of fossil canid skulls contradicts the suggested domestication of dogs during the late Paleolithic
Source: Sci Rep. 2015 Feb 5;5:8299. doi: 10.1038/srep08299 (PMC5389137; doi:10.1038/srep08299)
Supplement: Supplementary Information — Figure S1 [file srep08299-s2.pdf]

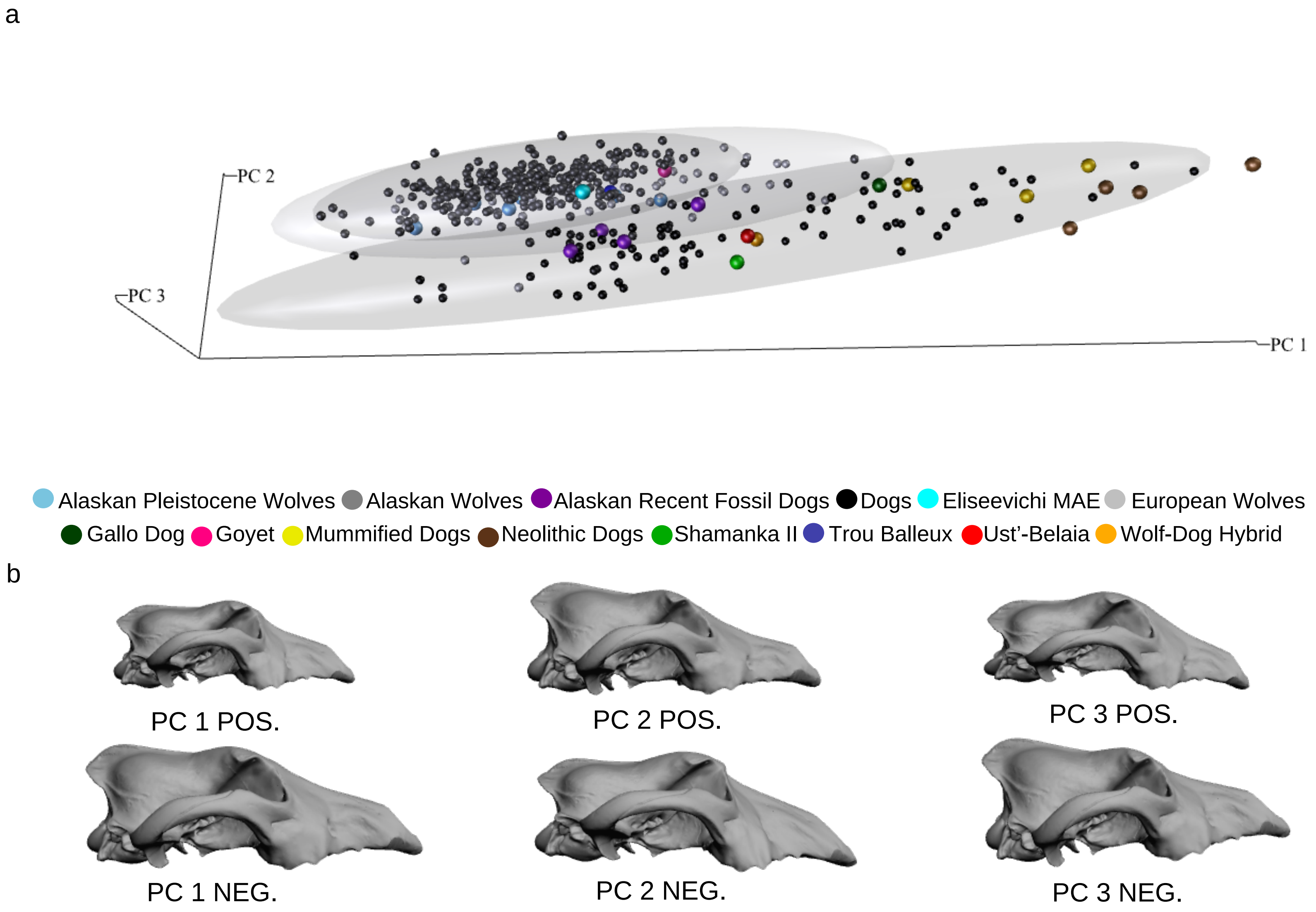
a
PC 2
PC 3
PC 1
Alaskan Pleistocene Wolves
Alaskan Wolves
Alaskan Recent Fossil Dogs
Dogs
Eliseevichi MAE
European Wolves
Gallo Dog
Goyet
Mummified Dogs
Neolithic Dogs
Shamanka II
Trou Balleux
Ust'-Belaia
Wolf-Dog Hybrid
b
PC 1 POS.
PC 2 POS.
PC 3 POS.
PC 1 NEG.
PC 2 NEG.
PC 3 NEG.
