# Supplementary figures and images for: 3D morphometric analysis of fossil canid skulls contradicts the suggested domestication of dogs during the late Paleolithic
Source: Sci Rep. 2015 Feb 5;5:8299. doi: 10.1038/srep08299 (PMC5389137; doi:10.1038/srep08299)

a

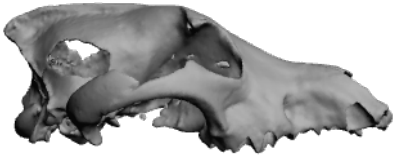

b

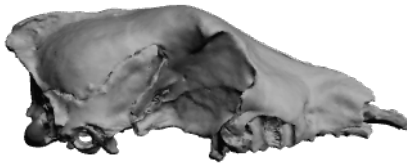

c

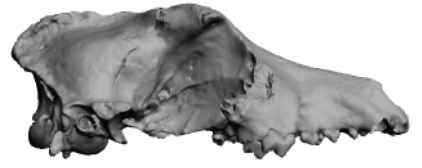

d

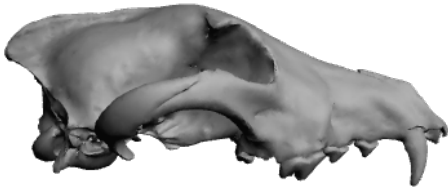

e

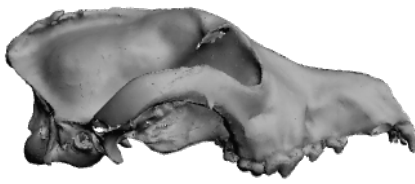

Supplement: Supplementary Information — Figure S2 [file srep08299-s3.pdf]

a

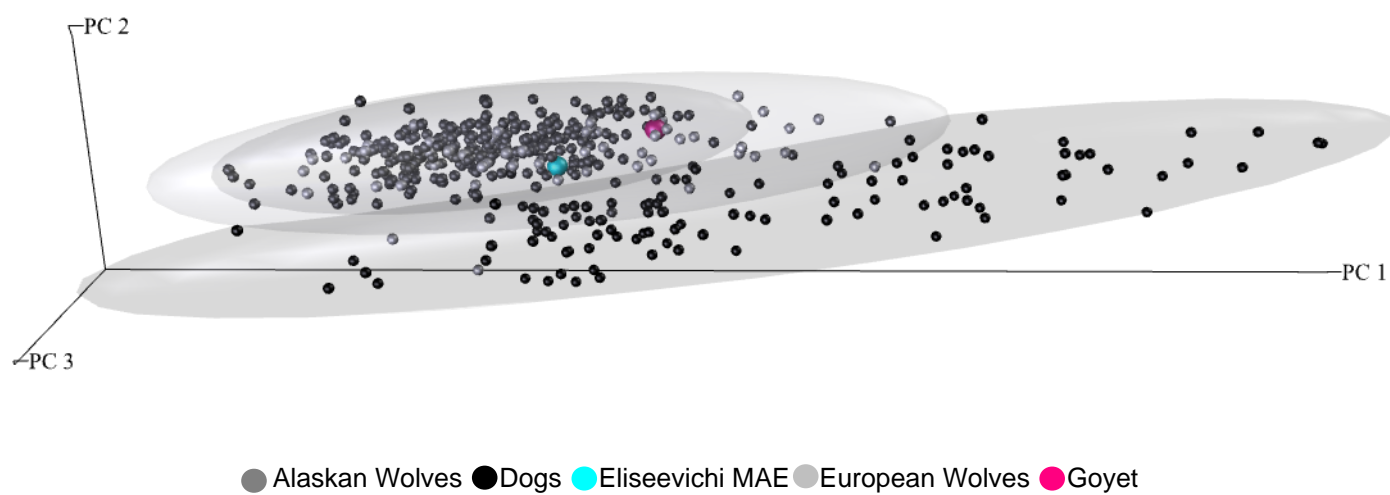

b

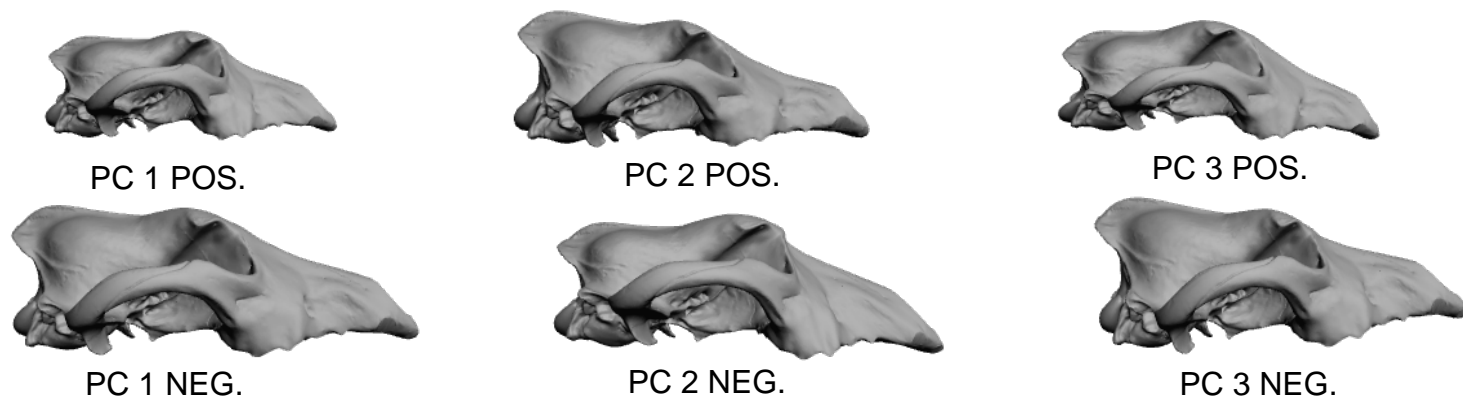

Supplement: Supplementary Information — Figure S3 [file srep08299-s4.pdf]
